# The response of the Military Health System (MHS) to the COVID-19 pandemic: a summary of findings from MHS reviews

**DOI:** 10.1186/s12961-023-01093-4

**Published:** 2024-01-08

**Authors:** Alysa Pomer, Satish Munigala, Christian L. Coles, Jessica Pope Mitro, Andrew J. Schoenfeld, Joel S. Weissman, Tracey Perez Koehlmoos

**Affiliations:** 1 Center for Surgery and Public Health, Brigham and Women’s Hospital, 1620 Tremont Street, Boston, MA 02120 United States of America; 2 Henry M. Jackson Foundation for the Advancement of Military Medicine, Bethesda, MD United States of America; 3 Uniformed Services University of Health Sciences, Bethesda, MD United States of America; 4 Department of Global and Community Health, George Mason University, Fairfax, VA United States of America; 5 Department of Orthopaedic Surgery, Brigham and Women’s Hospital, Boston, MA United States of America; 6 Department of Health Policy and Management, Harvard Medical School, Boston, MA United States of America

**Keywords:** COVID-19 pandemic response, Military Health System, Military health, Military medicine

## Abstract

**Introduction:**

The coronavirus disease 2019 (COVID-19) pandemic caused major disruptions to the US Military Health System (MHS). In this study, we evaluated the MHS response to the pandemic to understand the impact of the pandemic response in a large, national, integrated healthcare system providing care for ~ 9 million beneficiaries.

**Methods:**

We performed a narrative literature review of 16 internal Department of Defense (DoD) reports, including reviews mandated by the US Congress in response to the pandemic. We categorized the findings using the Doctrine, Organization, Training, Materiel, Leadership, Personnel, Facilities, and Policy (DOTMLPF-P) framework developed by the DoD to assess system efficiency and effectiveness.

**Results:**

The majority of the findings were in the policy, organization, and personnel categories. Key findings showed that the MHS structure to address surge situations was beneficial during the pandemic response, and the rapid growth of telehealth created the potential impact for improved access to routine and specialized care. However, organizational transition contributed to miscommunication and uneven implementation of policies; disruptions affected clinical training, upskilling, and the supply chain; and staffing shortages contributed to burnout among healthcare workers.

**Conclusion:**

Given its highly integrated, vertical structure, the MHS was in a better position than many civilian healthcare networks to respond efficiently to the pandemic. However, similar to the US civilian sector, the MHS also experienced delays in care, staffing and materiel challenges, and a rapid switch to telehealth. Lessons regarding the importance of communication and preparation for future public health emergency responses are relevant to civilian healthcare systems responding to COVID-19 and other similar public health crises.

**Supplementary Information:**

The online version contains supplementary material available at 10.1186/s12961-023-01093-4.

## Background

The COVID-19 pandemic caused global disruptions to societies, economies, and healthcare delivery. The initial severity of the pandemic forced governments and health systems to rapidly adopt a broad range of previously untried policies and practices. The United States implemented a “whole-of-government” approach to leverage a coordinated response to the pandemic across departments and agencies. While the Military Health System (MHS) was part of the whole-of-government response, as a large-scale healthcare delivery network, the MHS was also among the healthcare systems heavily impacted by the COVID-19 pandemic. The MHS delivers healthcare to 9.6 million service members, retirees, and their dependents [1]. This care is delivered via a two-pronged direct and private sector care system. Direct care is provided through more than 700 medical treatment facilities (MTFs), including 51 inpatient hospitals and medical centres, with an estimated 144,000 personnel in the military medical workforce [2]. Private sector care is provided via the TRICARE benefit, which functions as health insurance in the US civilian healthcare sector. The MHS is separate from the Veterans’ Administration (VA) health system.

The US Congress mandated a review of the MHS pandemic response during the 2021 fiscal year via section 731 of the National Defense Authorization Act (NDAA) [3]. In response, the DoD submitted a report to Congress [4]. In this review, we summarize the report to Congress and other reports initiated by the DoD in response to the NDAA mandate to understand the impact of the COVID-19 pandemic on the MHS and the implications of the system’s pandemic response.

## Methods

We performed a narrative review of all existing internal DoD reports generated as a part of the Congressional-mandated review of the MHS response to the COVID-19 pandemic. The 16 original reports were published between November 2020 and December 2022 (Table 1). One of our authors (TK) was involved in the development of the Congressional-mandated review and provided all the reports used for this study. A narrative review was chosen as the most appropriate methodology for this review, as our purpose is to identify and summarize the existing literature but not to create new theories or analyse the findings in relation to existing theories [5, 6]. A key feature of the narrative review is that it does not involve a systematic search of the literature [5]; this team previously completed a systematic review of existing literature on this topic [7], so this narrative review provides an opportunity to explore the grey literature not included in that report and bring those resources into public domain.

**Table 1:** Studies included in the analysis (sorted by year)

Title	Organization	Year
Rapid Environmental Scan of the United States’ Health Services System Surge Capacity in Support of the Military Health System	Uniformed Services University of the Health Sciences	2020
Financial impact	Institute for Defense Analyses	2021
Medical force structure and manning	Institute for Defense Analyses	2021
Global health engagement and security activities	Institute for Defense Analyses	2021
Governance and organization	Institute for Defense Analyses	2021
Logistics and technology	Institute for Defense Analyses	2021
Medical education and training	Institute for Defense Analyses	2021
Operational capabilities and support	Institute for Defense Analyses	2021
Policy	Institute for defense analyses	2021
Public health	Institute for Defense Analyses	2021
Research, diagnostics, and therapeutics	Institute for Defense Analyses	2021
TRICARE	Institute for Defense Analyses	2021
Query on Staffing Advocacy as Reported in the MHS COVID-19 Lessons Learned Database	Institute for Defense Analyses	2021
NDAA 2021, section 731: Novel COVID-19 Virus After Action Report	TRICARE Working Group, US Department of Defense	2021
Evaluation of Department of Defense Military Medical Treatment Facility Challenges During the Coronavirus Disease-2019 (COVID-19) Pandemic in Fiscal Year 2021	Office of the Inspector General of the US Department of Defense	2022
Report to Congressional Defense Committees: COVID-19 Military Health System Review Panel	US Department of Defense	2023

**Fig. 1:**
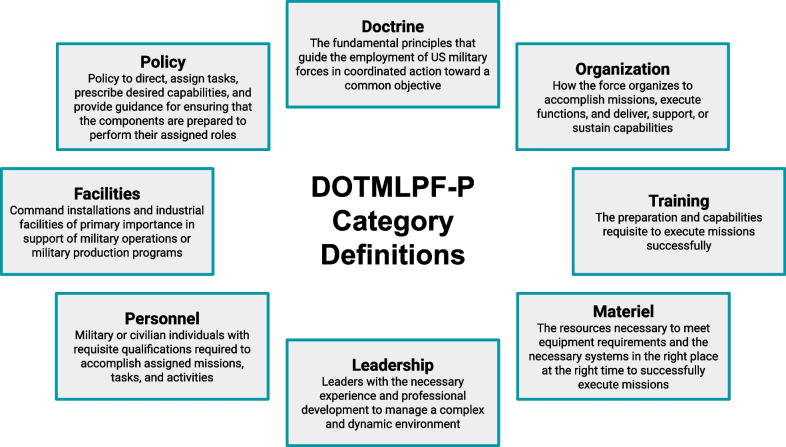
DOTMLPF-P framework category definitions

All reports were reviewed by two authors (A.P., S.M.), and the findings were organized using the Doctrine, Organization, Training, Materiel, Leadership, Personnel, Facilities, and Policy (DOTMLPF-P) framework developed in the DoD [8]. In the majority of the DoD reports, findings were explicitly listed; where findings were not explicitly listed, two authors (A.P., S.M.) evaluated the report and came to a consensus on findings. The DOTMLPF-P framework definitions are illustrated in Fig. 1. This framework is used in the Joint Capabilities Integration Development System process and is designed to address leadership-defined capability shortfalls or gaps. Each report finding was assigned to a single category determined to be the best fit. Final agreement on categorization of the findings was by consensus of four authors (A.P., S.M., C.C., J.P.M.).

## Findings

**Fig. 2:**
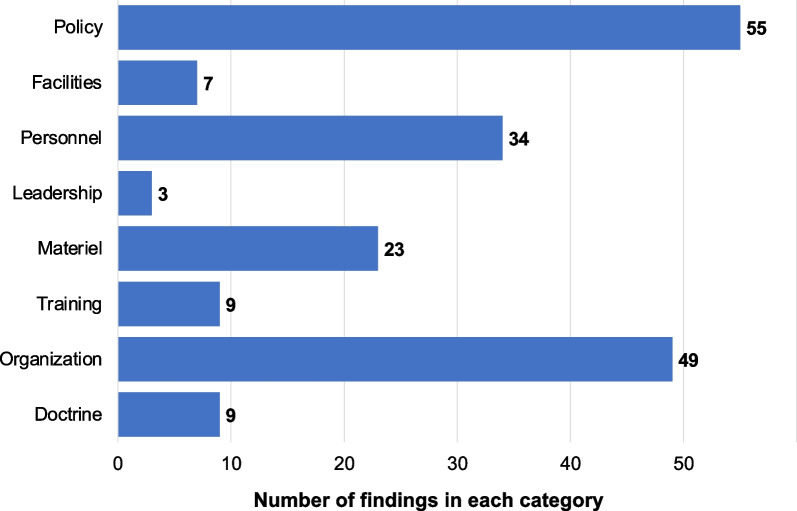
Findings by DOTMLPF-P category

A total of 189 findings were identified across the eight DOTMLPF-P categories, with the majority of findings categorized under policy (55), organization (49), and personnel (34) (Fig. 2). A complete list of findings is available in Additional file 1. Below we summarize the findings within each category.

### Doctrine

Doctrine refers to the fundamental principles that guide the employment of US military forces in coordinated action toward a common objective. There were nine findings in the doctrine category.

COVID-19 illness and pandemic restrictions disrupted MHS missions [9, 10], and running the COVID-19 healthcare delivery mission concurrent with pre-existing missions created competition for staff and resources [9]. Several COVID-19-specific missions evolved, including supporting a range of civil authorities in vaccine treatment and development (such as Operation Warpspeed) and deploying MHS medical personnel to support civilian medical systems. DoD’s in-house scientific expertise in vaccine and treatment development and high-containment pathogens was instrumental in pandemic-related research informing these missions and civilian responses [4]. This ability to quickly respond to changing circumstances and to utilize existing in-house expertise illustrates how the MHS system could serve as a model for other large healthcare systems [11].

### Organization

Organization is how the force organizes to accomplish missions, execute functions, and deliver, support or sustain capabilities. There were 49 findings in the organization category.

Many findings under this category related to the major organizational transition that the MHS was undergoing as the pandemic began. This transition involved consolidating the health services of the Army, Navy and Air Force under a single Defense Health Agency; this consolidation process began in October 2018 and was scheduled to be completed by October 2021 [2]. While the incomplete transition contributed to unclear communication and chains of command, which created duplicated efforts and planning gaps in the MHS’ COVID-19 response [4, 7, 9, 12, 13], the pandemic also impeded progress in implementing parts of the organizational transition [4].

During the pandemic, concerns emerged around eligibility for covered services, including who could receive COVID-related healthcare through the MHS and where beneficiaries should receive care. For example, to protect service members, consideration for medical needs was given to individuals who may not have been eligible for MHS benefits but who worked closely with service members, such as contractors and civilian employees [4]. This increased burden led to insufficient capacity to balance pandemic response missions in the MHS beneficiary population with response missions in the civilian community [4]. For MHS beneficiaries, there was increased reliance on TRICARE as a primary health insurance provider and increased outsourcing to the civilian medical sector due to staffing issues at military treatment facilities [9, 14].

Despite these challenges, the MHS COVID-19 pandemic response benefitted from the centralized structure of the MHS, as it meant COVID-19 guidelines could be issued and implemented for the whole organization to create a uniform response [7]. This structure also allowed MHS systems to quickly scale up and increase diagnostic testing capacity at research laboratories [15], maintain a blood program that could adapt to quickly changing recommendations for convalescent plasma treatment [4] and utilize robust biosurveillance systems to identify and rapidly respond to infectious disease threats for COVID-19 and any potential future disease threats [15, 16].

### Training

Training is the preparation and capabilities required to execute missions successfully. There were nine findings in the training category. The pandemic led to disrupted clinical learning, resulting in a cohort of recently-graduated students entering the MHS as trainees (e.g. residents) or new providers with non-typical and variable medical education experiences and fewer opportunities to interact with patients than in previous cohorts. For example, the Uniformed Services University of the Health Sciences suspended clinical rotations and graduated fourth year medical students early during the first months of the pandemic to aid at MTFs; other medical schools similarly graduated medical students early to work in overwhelmed hospitals, but some medical schools instead delayed graduations during this time [17]. These new medical professionals will likely require additional training and supervision in the early stages of their careers [4, 18]. However, many of these trainees and new providers have increased telehealth experience due to the shift to telehealth during the pandemic; as a result, these cohorts may be more likely to continue providing telehealth options for MHS beneficiaries [18]. Classroom curricula also changed during the pandemic, with an increased focus on public health issues that gained prominence during the pandemic, such as access to care, food and housing insecurity, and racial disparities in care and outcomes [18]. Medical staff experienced decreased ability to stay current with clinical skills as a result of reduced healthcare services at MTFs [9]. On the contrary, because MHS training already focuses on critical care and other skills crucial in a public health emergency, the MHS has the capacity to be a leader in future public health emergencies by ensuring that crisis training programs exist to quickly right skill and up skill staff [14].

### Materiel

Materiel is the resources necessary to meet equipment requirements and the necessary systems in the right place at the right time to execute missions successfully. There were 23 findings in the materiel category.

While personal protective equipment (PPE) and pandemic-related equipment stocks were adequate for MHS requirements, the whole-of-government response to the pandemic made additional demands on these by requiring equipment sharing with entities outside the MHS [4]. Additionally, DoD public emergency policies lacked clarity on ownership and release authority for PPE, and the military medical supply chain relied heavily on China for medical materiel, making it difficult to resupply depleted stock [4]. One report suggested that better use of data and analytics may mitigate future shortages by increasing supply chain resiliency and by improving planning for situations in which supply chains are disrupted longer than the time it takes to deplete stockpiles [19]. Supply chain issues also contributed to discontinuities with vaccine administration, with delays in shipping vaccine supplies. This compounded vaccine distribution and administration issues caused by a lack of a single cohesive plan for vaccine administration across MTFs, and misalignment between distribution and demand [4].

Pre-existing shortages of IT equipment left MHS under-equipped to transition to a virtual environment. Additional capability to conduct remote healthcare was hindered because remote health applications were not initially approved, and there were challenges with bandwidth and access to the virtual private network. The move to a virtual environment was also complicated by the in-progress MHS transition, which meant not all IT infrastructure was under the consolidated network, instead falling under different authorities [4].

Emerging data technologies, such as those designed to improve patient-condition-change warnings, may be useful for managing risk and mortality [16]. The planned rollout of the new electronic health record (EHR) for the MHS was delayed [7]; however, in locations where the new EHR had been activated prior to the start of the pandemic, a COVID-19-specific communication tool was created [7]. At the population level, the MHS could leverage its big data capabilities to advance disease surveillance where traditional measures (such as case counts and testing rates) are not available or sufficient [11].

### Leadership

Leadership is having leaders with the necessary experience and professional development to manage a complex environment. There were three findings in the leadership category. These findings demonstrated that clear and consistent lines of authority, responsibility and accountability needed to be reinforced [12], and an insufficient number of trained personnel were available to fill key leadership roles in the pandemic response [7]. Compounding these issues, delayed and cancelled care was more likely to affect beneficiaries over the age of 40 years, which includes 85% of senior officers [20].

### Personnel

Personnel is the military or civilian individuals with requisite qualifications to accomplish assigned missions, tasks and activities. There were 34 findings in the personnel category. In early reports, personnel concerns primarily centred around insufficient staffing due to staff illness and exposure, caretaking responsibilities, movement restrictions, increased demand for staff, insufficient specialized training, hiring freezes and reassignments or deployments, all of which compounded existing staffing shortages [4, 7, 10, 14, 21]. Early reports also predicted staff burnout and mental health concerns would become a significant issue [14], a prediction supported in later reports and exacerbated by limited access to behavioural healthcare [4, 9]. Findings of staff shortages persisted in later reports, which were noted to be the most serious challenge faced at MTFs [9].

The MHS used several methods to adapt to staffing shortages, including hiring travel nurses and bringing back retired staff [14]. Recruiting and hiring new staff was challenging due to non-competitive salaries, lack of funding and slow hiring procedures [9], which are further detailed in the Policy section below. Future hiring outlooks were generally positive, as broad changes across the economy could lead to more hiring opportunities for the MHS [18], and expanded workplace flexibility may help retain existing employees and maintain capacity [22]. Additionally, developing and encouraging in-house research staff with interests beyond traditional military infectious disease health threats will enable rapid responses to future health threats [15].

### Facilities

Facilities are command installations and industrial facilities of primary importance in support of military operations or military production programs. There were seven findings in the facilities category. MTFs were not set up to minimize transmission risk and required environmental modifications to reduce the risk of COVID exposure among staff and patients [4]. Paradoxically, while decreased healthcare utilization created a surplus of beds at MTFs [7], decreased staffing at MTFs led to increased reliance on civilian healthcare, although beds were often unavailable in this sector [9]. Additionally, the research laboratories of the vertically structured MHS have the potential to rapidly expand the diagnostic testing base. In future public health emergencies, leveraging this capability could reduce testing bottlenecks [15].

### Policy

Policy is having the appropriate policies to direct, assign tasks, prescribe desired capabilities and provide guidance for ensuring that the components are prepared to perform their assigned roles. There were 55 findings in the policy category.

Updated policies led to increased telehealth visits [7], though behavioral health policies limited the use of telehealth for behavioural healthcare and treatment [13]. This created the potential to expand virtual capabilities worldwide across a broader range of patient services than many other healthcare systems for routine and specialized care [12, 22], though a universal strategy for telehealth has not yet been implemented [4]. TRICARE was slow to approve these options and clarify coverage [4], in part because TRICARE is subject to several regulatory constraints when changing or updating policy as significant changes require new legislation [23]. As an alternative to legislation, workarounds to make TRICARE policy changes involved working within the Defense Health Agency to find exemptions to existing laws and regulations and proactively identifying future additions and changes [13].

As noted in the Personnel section, personnel shortages were common, and several solutions were posited to mitigate the problems of personnel shortages. First, flexible workplace policies (including remote working) allowed the MHS to maintain capability and fulfil its organizational mission [10, 22]. Second, staffing shortages could be covered using civilian and contract personnel, as well as Guard and Reserve personnel [9]; however, the hiring process was slow and cumbersome, and spending policies for hiring civilian and contract staff created additional difficulties in hiring new personnel [9], making this option less feasible. Finally, noting the impact of strain on personnel mental health, policies to mitigate the circumstances which lead to personnel burnout need to be implemented for staff on and off MTFs, including improving hiring policies to ensure staff are not overburdened due to staffing shortages [9].

The third set of policy findings regarded health emergency preparedness policies. First, military medical research was crucial to developing and implementing containment strategies; building upon existing platforms for clinical practice allowed rapid development of COVID-19 treatment guidelines, as well as the capacity to quickly update and disseminate those guidelines [7]. Developing hotspot prediction systems to supplement civilian systems could create opportunities to build partner networks and develop inter-agency coordination [10, 16, 22]. However, funding and research quickly shifted away from non-COVID-related topics at the start of the pandemic, so some research that could have been beneficial in future planning may have been negatively affected [4].

The fourth set of policy findings involved control of COVID-19 within the DoD. Many of the issues encountered by the MHS originate from the known gap in public health command and coordination for the whole enterprise at the start of the pandemic, meaning that crucial policies for responding to a public health event, such as a contact tracing plan or a tracking and reporting system, had not been established [4]. Additionally, local-level commanders were given latitude to adjust responses on the basis of local conditions, so mitigation guidance and pandemic responses were uneven and varied [4]. This was particularly relevant in overseas installations, where international guidelines differed from and sometimes contradicted MHS guidelines, creating cultural and accessibility difficulties [4]. MHS also encountered legal and Health Insurance Portability and Accountability Act (HIPAA) privacy challenges domestically in the process of administering vaccines [4].

## Discussion

From these 16 reports, we identified 189 findings across 8 categories of the DOTMLPF-P framework. These findings highlighted the benefits of the structure of the MHS to support the surge in need for clinical care experienced in the early phases of the COVID-19 pandemic. The findings showed the negative impact of the incomplete MHS organizational transition on creating clear communication and policy implementation. The findings also showed an increased and expanded use of telehealth in the MHS and increased reliance on civilian sector services instead of direct care. Finally, these findings illustrated disrupted clinical training and supply chains, as well as staffing shortages and burnout among MHS healthcare workers.

The findings on the impacts of the COVID-19 response in the MHS echo experiences elsewhere in the US health system and in other global healthcare networks. For example, Balser et al. [24], found that partnerships with organizations across US health systems were crucial to supporting public health aspects of pandemic care. The MHS response was defined by some of those partnerships as it engaged in the whole-of-government response and other civilian support missions; Koehlmoos et al. [25] and Goralnick et al. [26], both acknowledge the importance of such civilian–military partnerships beyond disaster and emergency situations to increase healthcare system resiliency. El Bcheraoui et al. [27], Haldane et al. [28] and Narain et al. [29] observed how pandemic responses exposed weaknesses in health systems worldwide and demonstrated how health systems work within greater health, social and economic structures; the MHS likewise saw system weaknesses exposed by the pandemic response alongside new awareness as to how these weaknesses may be improved, largely in organization and policy findings. Kendzerska et al. [30] found that an increase in telehealth services may help alleviate future disruptions and increase access, similar to the MHS experience with telehealth during the pandemic. However, Balser et al. [24] found that, like in the MHS, civilian health system operations were impacted by inadequate materials and staffing resources, many of which were previously existing issues that were exacerbated by the pandemic. Berlin et al. [31] and Popowitz [32] both show how the civilian healthcare workforce has experienced significant negative impacts as a result of the pandemic and the healthcare system’s response to the pandemic which exacerbated an already tenuous situation; similarly, MHS reports consistently demonstrate the pandemic’s negative impacts on MHS healthcare workers, indicating that personnel is a continuing challenge for the MHS. Because the difficulties faced by the MHS are similar to many of the difficulties faced by civilian health systems during the pandemic, this indicates that some of the solutions employed by the MHS may also benefit other systems in future public health emergencies, such as building flexibility into policy and planning and developing and nurturing in-house expertise.

A limitation of this work is that each finding is sorted under a single category for simplicity; it is worth noting that many findings could be classified under more than one category. Most of these reports were generated during the first year of the COVID-19 pandemic, thus the impact of successive waves and new variants is unclear, and the reports do not reflect trends of continued stress on systems and personnel. However, the most recent reports [4, 9] indicate that many of the policy and organizational challenges became less significant in year 2 of the pandemic, and challenges around materiel declined. At the same time, personnel challenges increased and became more urgent, although the exact nature of these challenges varied across the timeframe of the pandemic.

A strength of our study is the use of internal DoD reports and documents that were generated as a part of the 2021 NDAA that mandated a review of the MHS pandemic response. Although these documents would not ordinarily be included in scientific analyses, their use here will bring these reports and their results into the peer-reviewed literature. Another strength is the use of the DOTMLPF-P framework, which can be used to evaluate health systems, similar to the World Health Organization’s Building Blocks of Health Systems framework [33], but benefits from being more familiar to leaders in the DoD and MHS.

## Conclusion

The results of the DoD-initiated reports on the MHS response to the COVID-19 pandemic suggest that the MHS was less than fully prepared to provide an immediate and robust response to the COVID-19 pandemic. The MHS experienced miscommunications, delays in care and personnel challenges, similar to many of the problems seen in civilian healthcare systems. At the same time, the unique structure of the MHS placed it in a better position than many civilian healthcare networks to respond quickly to the pandemic and prepare for future public health emergencies. These lessons, which underline the importance of communication and preparation, are relevant to civilian healthcare systems responding to COVID-19 and other similar public health crises in the future.

## Disclaimer

The contents of this publication are the sole responsibility of the authors and do not necessarily reflect the views, assertions, opinions or policies of the Uniformed Services University of the Health Sciences (USUHS), the Henry M. Jackson Foundation for the Advancement of Military Medicine, Inc. (HJF), the Department of Defense (DoD) or the Departments of the Army, Navy or Air Force. Mention of trade names, commercial products or organizations does not imply endorsement by the US Government.

### Supplementary Information

**Additional file 1.** A table listing all findings from reports in the study, organized by DOTMLPF-P category.

## Data Availability

Not applicable.

## Abbreviations

DoD: Department of Defense
DOTMLPF-P: Doctrine, Organization, Training, Materiel, Leadership, Personnel, Facilities, and Policy framework
MHS: Military Health System
MTF: Medical treatment facility
NDAA: National Defense Authorization Act
PPE: Personal protective equipment
