# Correction: The response of the Military Health System (MHS) to the COVID-19 pandemic: a summary of findings from MHS reviews

**DOI:** 10.1186/s12961-024-01109-7

**Published:** 2024-01-30

**Authors:** Alysa Pomer, Satish Munigala, Christian L. Coles, Jessica Pope Mitro, Andrew J. Schoenfeld, Joel S. Weissman, Tracey Perez Koehlmoos

**Affiliations:** 1 Center for Surgery and Public Health, Brigham and Women’s Hospital, 1620 Tremont Street, Boston, MA 02120 USA; 2 Henry M. Jackson Foundation for the Advancement of Military Medicine, Bethesda, MD USA; 3 Center for Health Services Research, Uniformed Services University of Health Sciences, Bethesda, MD USA; 4 Department of Global and Community Health, George Mason University, Fairfax, VA USA; 5 Department of Orthopaedic Surgery, Brigham and Women’s Hospital, Boston, MA USA; 6 Department of Health Policy and Management, Harvard Medical School, Boston, MA USA

**Correction: Health Research Policy and Systems (2024) 22:5** 10.1186/s12961-023-01093-4

Following publication of the original article [1], it was reported that Tracey Perez Koehlmoos was erroneously listed as being affiliated with affiliation 2 in addition to affiliation 3. The correct affiliations are given in this Correction and the original article has been updated.
