# Supplementary material for: The response of the Military Health System (MHS) to the COVID-19 pandemic: a summary of findings from MHS reviews
Source: Health Res Policy Syst. 2024 Jan 8;22:5. doi: 10.1186/s12961-023-01093-4 (PMC10773054; doi:10.1186/s12961-023-01093-4)
Supplement: Supplementary file 1 — Additional file 1. A table listing all findings from reports in the study, organized by DOTMLPF-P category. [file 12961_2023_1093_MOESM1_ESM.docx]

**Additional file 1: All report findings, organized by DOTMLPF-P category**

| **Doctrine** | | |
| --- | --- | --- |
| *Report Name* | *Finding* | *page* |
| IDA Report: Global Health Engagement and Security Activities | COVID restrictions affected global missions | 3 |
|  | Partner capability within specific activities can help mitigate the effect of disruptions on Global Health efforts | 11 |
| IDA Report: Public Health | The Department of Defense can be a leading example to the public in pandemic response | 8-9 |
| DOD Inspector General Report | MTFs’ COVID-19 pandemic response competed with the MTFs’ health care delivery mission for staff | 7 |
|  | The national COVID-19 pandemic response and global missions competed with the MTFs’ health care delivery mission for MTF staff | 8, 9 |
|  | As a result of the personnel shortages and staff burnout, officials reported that MTFs faced staff resignations, and MTF patients encountered delays in receiving care and increased safety risks | 7 |
| DOD Report to Congress | A percentage of the MHS-covered population chose not to receive the vaccine | 8 |
|  | DoD in-house scientific expertise was instrumental in pandemic-related research | 10 |
|  | MHS provided a range of support to civil authorities | 11 |
|  | Doctrinal and operation roles for DHA in the DoD response for COVID-19 was unclear | 12-13 |

| **Organization** | | |
| --- | --- | --- |
| *Report Name* | *Finding* | *page* |
| IDA Report: Financial Impact | Delayed and canceled care may lead to higher costs | 7 |
|  | COVID-19 pandemic-induced job loss for retirees and dependents may shift costs to TRICARE | 8 |
| IDA Report: Force Structure and Manning | MHS structure is ideal for surge scenarios | 9 |
|  | Civilian surge capacity is limited | 9-10 |
| IDA Report: Global Health Engagement and Security Activities | Enhance global capacities to prevent, detect, and respond to infectious disease threats and global health engagement to improve health system capacity | 4 |
|  | Operational flexibility and adaptability were key to continuing progress toward mission goals during disruptive events | 10 |
| IDA Report: Governance and Organization | Common themes for delivering robust virtual care emerged across health systems: well-defined triage rules, dedicated materiel for virtual care, and appropriate measures of performance | 3 |
|  | Reinforce clear and stable lines of authority, responsibility, and accountability | 10 |
| IDA Report: Logistics and Technology | Poorly managed stockpiles lead to supply shortfalls and delivery of unusable supplies | 6-7 |
|  | Data and analytics can help allocate scarce resources, but cannot replace building and maintaining surge production capacity | 9 |
| IDA Report: Operational Capabilities & Support | If the MHS relies on civilian early warning biosurveillance systems, the MHS will inherit the limitations of the civilian systems | 8 |
| IDA Report: Policy | Expanded workplace flexibility can maintain capability and fulfill organizational mission | 7 |
| IDA Report: Research, Diagnostics, & Therapeutics | Maintaining a knowledge base on potential infectious disease threats can contribute to combatting the next emerging disease | 6-7 |
|  | Being ready to broaden diagnostic testing base for rapid expansion may aid in medical readiness | 7-8 |
| TRICARE Working Group AAR | Wide range of communications tactics were employed to inform beneficiaries about health care changes | 1 (Executive Summary),  3-4 (Full Report) |
| DOD Inspector General’s Report | Lack of coordination between the Services and the DHA about personnel diversion | 8 |
|  | Improvement suggestion: Establish unity of command or unity of effort. | 14 |
|  | DoD military MTFs continue to face enduring COVID-19 pandemic challenges | 32, 33 |
|  | Improvement suggestion: Reassess the TRICARE network. | 15 |
| USU Rapid Environmental Scan | Centralized nature of MHS (vs civilian health sector) permitted creation of pandemic guidelines | 4 |
|  | There was a need for a more coordinated DSCA response | 8 |
|  | Where the MHS encountered financial challenges during COVID-19, these challenges were often due to confusion around funding responsibilities | 14 |
| DOD Report to Congress | MHS was not prepared to immediately respond to a pandemic as the medical structure is designed for wartime | 7 |
|  | DoD labs provided support for epidemiological testing and research, despite a gap in organic capability to do field tests and research | 10 |
|  | DoD deployed multiple assets for surge medical support to support civil authorities | 11 |
|  | MHS deployed personnel to support civil authorities in administering vaccinations | 11 |
|  | DoD capacity for medical aid to assist civilian authorities is limited due to increased internal requirements for medical care | 11 |
|  | Some deployed medical capabilities saw minimal or no utilization by civil authorities, resulting in depleted availability for other requests from civil partners | 11 |
|  | The medical capacity of the DoD was greatly augmented by National Guard assets to support civil authorities | 11 |
|  |  |  |
|  | Investments in medical intelligence capabilities were not adequate to meet the demands of the fast-moving event | 11 |
|  | The Armed Services Blood Program quickly adapted to emerging needs | 12 |
|  | The incomplete transition to DHA at the start of the pandemic resulted in significant issues in doctrine, guidance, authorities, and unity of command | 12 |
|  | Response required application of significant clinical capabilities integrated with Service responsibilities over operational medical forces | 13 |
|  | The pandemic impeded progress in implementing the public health transition | 13 |
|  | Effective communication and collaboration among MHS component C2 elements were uneven and inadequate | 13 |
|  | The absence of an authoritative source for data, analysis, and modeling resulted in confusion | 13 |
|  | External partners impose significant expectations and demands on DoD to provide critical operational capability in response to national emergency | 13 |
|  | The coordination of medical DSCA operations was less than optimal at multiple levels | 13 |
|  | There is insufficient capacity within the health care system for a pandemic response to balance support with DoD beneficiary population and additional community support | 13 |
|  | National Guard (NG) mobilizations successfully satisfied most assistance requests tendered by civil authorities | 13 |
|  | Effective communication among MHS component pandemic C2 elements was not adequate or consistent | 14 |
|  | The rapid initiation of new R&D in response to COVID-19 led to deviation from well-established requirements-based processes | 14 |
|  | Previous research informed DoD’s understanding of the threat and enabled swift initiation of countermeasures and treatment plans | 14 |
|  | Global public health and biosurveillance communities were not well integrated or funded to address the threat posed by disease and biological agents | 16 |
|  | Difficulties continue in implementing multi-year GHE plans | 16 |
|  | The domestic military response evolved to include four medical concepts of operations, of which embedding was found to be the most effective | 17 |
|  | Combatant commands and military departments were able to quickly adjust the way in which forces were organized and employed | 17 |
|  | Operations focused on continental US installations and were sometimes unable to quickly adapt to challenges of outside continental US installations | 17 |
|  | To protect the Armed Forces and ensure operational readiness, consideration was given to those individuals with whom Service members regularly come into contact in a work environment, even though they were not eligible MHS beneficiaries | 19 |

| **Training** | | |
| --- | --- | --- |
| *Report Name* | *Finding* | *page* |
| IDA Report: Force Structure and Manning | MHS has an opportunity to be a leader in pandemic right-skilling and up-skilling | 10 |
| IDA Report: Medical Education and Training | Clinical learning was disrupted, but new learning experiences were created | 4 |
|  | Curriculum change reflected changes in public health priorities | 5 |
|  | Student experiences and behavior was disrupted across the medical education and training pipeline | 6 |
|  | MHS will face a cohort of medical accessions with non-typical and variable medical education experiences | 7 |
|  | New MHS accessions will have more telehealth experience | 7 |
| DOD Inspector General’s Report | Staffing shortages led to reduced staff training | 7, 12 |
| DOD Report to Congress | COVID-19 adversely affected recruiting, accession, and entry-level training activities | 16 |
|  | DoD successfully continued accessions and initial training process by applying lessons learned in real time | 16 |

| **Materiel** | | |
| --- | --- | --- |
| *Report Name* | *Finding* | *page* |
| IDA Report: Logistics and Technology | Better access to data and analytics can increase supply chain resiliency | 6 |
|  | Plans need to include situations where supply chains are disrupted for longer than stockpiles last | 8 |
| IDA Report: Operational Capabilities & Support | Emerging tools designed to improve warning of patient condition changes may be useful to MHS providers for managing risk and mortality. | 8 |
| IDA Report: Public Health | MHS could leverage big data capabilities to advance force health protection and disease surveillance | 8 |
| USU Rapid Environmental Scan | Planned rollout of new EHR was delayed | 15 |
|  | COVID-19-specific communication tool was created in new EHR system | 16 |
| DOD Report to Congress | Various inputs and guidance around vaccines presented challenges to timely implementation and execution | 7 |
|  | Consistent vaccine documentation and integrity across MHS information technology systems was a challenge | 8 |
|  | Delays in shipping vaccine supplies caused discontinuities in vaccine administration | 8 |
|  | Vaccine distribution was misaligned with demand signal | 8 |
|  | There was not a single, cohesive plan for vaccine administration across MTFs | 8 |
|  | Stocks of PPE and pandemic-related equipment were adequate for MHS requirements, but additional demands were put on the stocks by outside responses | 9 |
|  | Military medical supply chain relies heavily on China for medical material | 9 |
|  | The broad increased need for telemedicine and the subsequent expansion had a positive response from providers and patients | 9 |
|  | MTFs experienced preexisting shortages of IT equipment, leaving the enterprise under-equipped to transition to a virtual environment | 11 |
|  | MTFs were in the middle of a transition from Service to DHA ownership, complicated because not all IT infrastructure fell under the same authority | 12 |
|  | Remote health applications were not yet approved, challenges with bandwidth, and access to the virtual private network caused inadequate capability to conduct virtual health care | 12 |
|  | DoD and civilian blood agencies experienced critical shortages of consumables and donors as the pandemic progressed | 12 |
|  | The Armed Services Blood Program was well-positioned to run a coordinated multi-Service operation for use of convalescent plasma | 12 |
|  | Policy for allocation of medical materiel was inconsistently executed across the system | 14 |
|  | MHS initially lacked a task force or designated lead to synchronize MHS-wide medical materiel management | 14 |
|  | DoD public emergency policies were not clear on ownership and release authority for PPE | 14 |
|  | Previous DoD investments in testing and evaluation technologies accelerated therapeutic discovery to inform clinical practice guidelines | 18 |

| **Leadership** | | |
| --- | --- | --- |
| *Report Name* | *Finding* | *page* |
| IDA Report: Financial Impact | Delayed and canceled care may lead to higher costs, especially for older individuals, which includes most high-level officers | 7 |
| IDA Report: Governance and Organization | Reinforce clear and stable lines of authority, responsibility, and accountability | 7 |
| USU Rapid Environmental Scan | Not enough trained personnel were available to fill key pandemic response leadership roles in MHS | 8 |

| **Personnel** | | |
| --- | --- | --- |
| *Report Name* | *Finding* | *page* |
| IDA Report: Force Structure and Manning | Facilities facing workforce shortages used multiple methods to build surge capacity, but these methods were not always enough | 5-6 |
|  | Healthcare capacity constraints was primarily about staff shortages | 6 |
|  | MHS worker mental health/burnout is a risk, and the inclusiveness and efficacy of current efforts is unknown. | 10 |
| IDA Report: Global Health Engagement and Security Activities | Operational capability impacted due to personnel illness | 2-3 |
| IDA Report: Medical Education and Training | Trainees with altered job prospects could present potential accession opportunities for DoD | 7 |
| IDA Report: Policy | Expanded workplace flexibility can maintain capability and fulfill organizational mission | 7 |
| IDA Report: Public Health | More research may be required on purchased care market performance | 9 |
| IDA Report: Research, Diagnostics, & Therapeutics | Staff with a broad knowledge base will aid in making rapid transitions and serving as experts for future emerging diseases | 6-7 |
| IDA Staffing Memo | 15 of 124 lessons learned referred to sufficiently staffed situations | 2 |
|  | 3 of 124 lessons learned referred to overstaffed situations | 2 |
|  | 106 of 124 lessons learned referred to understaffed situations | 2 |
| DOD Inspector General’s Report | Officials reported staffing and manpower shortages as the most serious challenge encountered by medical personnel at the MTFs during the COVID-19 pandemic | 6, 15 |
|  | Staff burnout due to personnel shortages and operational tempo is a challenge | 8, 12-13, 23 |
|  | Recruiting staff was a challenge | 6-7, 9 |
|  | Significant mental health concerns for staff remain | 23, 25 |
|  | Staff access to behavioral health care was limited | 23, 25 |
|  | Improvement suggestion: Increase MTF staffing | 26 |
|  | Improvement suggestion: Increase staffing for behavioral health services | 26 |
|  | Impact of staffing shortages: Reduced health care services at the MTF | 11 |
|  | Impact of staffing shortages: Delayed medical care | 12 |
|  | Impact of staffing shortages: Increased risk to patient safety | 13 |
|  | MTF officials also reported that a lack of funding inhibited their ability to hire more civilian and contract personnel in FY 2021 | 16 |
| USU Rapid Environmental Scan | The US military provided support to civilian hospitals in the form of providers during the COVID-19 crisis | 7-8 |
|  | While the MHS provided support to civilian healthcare, it also required support of its own | 8 |
| DOD Report to Congress | There were initial shortages in personnel trained for vaccine administration and documentation | 8 |
|  | Deployment of uniformed personnel from MTFs created gaps resulting in reduction of clinical and public health services | 15 |
|  | Stress on the medical system included limited ability for personnel to transfer and significant interruptions in specialty medical treatment | 15 |
|  |  |  |
|  | The ability of MHS to rapidly identify and mobilize medical professionals in support of an unplanned contingency was stressed | 15-16 |
|  | The pandemic highlighted risks associated with the differences in the way that Services report unit readiness for medical force elements | 16 |
|  | DoDI 6200.03: Section 5 (Surge Capabilities and Procedures for Health Care in DoD Public Health Emergencies) tasks MTFs with identifying resources to meet surge demands | 16 |
|  | Medical specialties in highest demand from the civilian sector were many of the same the specialties required for warfighting, which were chronically short in the DoD | 16 |
|  | There were insufficient number of public health emergency management personnel and resources at the outset of the pandemic | 17 |
|  | Public health emergency officials were required to be clinicians, some served multiple installations, and the role was a collateral duty on top of other responsibilities, creating a complicated workload | 17 |
|  | Within the MTFs there were serious personnel challenges stemming from personnel burnout, which were greatest in emergency departments and urgent care clinics | 17 |

| **Facilities** | | |
| --- | --- | --- |
| *Report Name* | *Finding* | *page* |
| IDA Report: Research, Diagnostics, & Therapeutics | Being ready to broaden diagnostic testing base in the event of future disruptive events | 7-8 |
| DOD Inspector General’s Report | Impact: Reliance on civilian system often meant beds were unavailable | 12 |
| USU Rapid Environmental Scan | Decreased healthcare utilization led to MTF beds being freed up | 12-13 |
| DOD Report to Congress | Physical space and design of DoD and VA healthcare facilities created challenges for controlling the spread of an infectious agent | 9 |
|  | MHS was able to develop near-immediate diagnostic testing capacity using legacy instruments compatible with the CDC’s national network | 10 |
|  | In certain areas outside of the continental US, MTFs were the sole authorized facilities for personnel testing | 10 |
|  | Clinical laboratories in MTFs and supporting research laboratories lacked infrastructure, capabilities, and materials to respond rapidly to pandemic-level testing needs | 10 |

| **Policy** | | |
| --- | --- | --- |
| *Report Name* | *Finding* | *page* |
| IDA Report: Force Structure and Manning | State regulations: Regulatory barriers were removed to expand the civilian medical workforce | 7 |
| IDA Report: Global Health Engagement and Security Activities | Operational flexibility and adaptability allow continued progress toward mission goals | 13 |
|  | Investigate opportunities for partner capability within specific activities | 14 |
| IDA Report: Governance and Organization | As a worldwide military support organization, the MHS has a unique opportunity to apply and expand virtual capabilities across a broader range of patient services than most healthcare systems | 9 |
| IDA Report: Operational Capabilities & Support | Earlier emerging hotspot identification may be valuable to the MHS in both of its roles, allowing for greater ability to avoid localized supply gaps | 8 |
| IDA Report: Policy | Expanded workplace flexibility can maintain capability and fulfill  organizational mission | 7 |
|  | Use of telehealth can improve access to routine and specialized care | 7-8 |
|  | National preparedness effort presents opportunity to enhance interagency coordination | 8 |
| IDA Report: Research, Diagnostics, & Therapeutics | Early planning for clinical trial management | 5-6 |
| IDA Report: TRICARE | TRICARE is subject to additional acquisition hurdles, lagging CMS and other private insurance providers in implementing emergency policy changes | 9 |
|  | Emerging COVID-19 digital engagement best practices may be worth investigating for application within TRICARE | 9 |
| TRICARE Working Group AAR | Access to telehealth use in behavioral healthcare was defined by the treatment program | 1 (Executive Summary),  6 (Full Report) |
|  | TRICARE benefit regulations should be updated to ensure they meet current standard of care | 7 |
|  | If congressional change is not possible, there are alternative methods through DHA to make changes to TRICARE | 7 |
| USU Rapid Environmental Scan | Building upon existing Joint Trauma System platforms permitted development of MHS clinical practice guidelines for treatment of COVID-19 | 4 |
|  | During a public health emergency, military medical research can be critical to containment, as is the case for the current COVID-19 pandemic | 5-6 |
|  | Updated telehealth policies caused an increase in telehealth encounters | 12 |
| DOD Inspector General’s Report | Improvement suggestion: Develop a manpower strategy for the personnel required for the COVID-19 mission | 14 |
|  | Hiring process is very long and cumbersome | 10, 25 |
|  | Improvement suggestion: Improve civilian and contract hiring. | 15 |
|  | Improvement suggestion: Create a strategy for fatigue and operational stress reduction. | 26 |
|  | Improvement suggestion: DoD should implement policies and practices to minimize the physical and psychological circumstances that lead to burnout for MTF staff | 27, 28 |
|  | DoD has policies or programs to reduce the psychological impacts of stress outside of MTFs, but not within MTFs | 29-30 |
|  | COVID-exposure policies contributed to staff shortages and overwork | 10 |
|  | Impact: Changes in MTF capabilities led to referrals to the civilian network, which affected readiness | 11-12 |
|  | Spending policies created hiring difficulties | 9 |
| DOD Report to Congress | Timely guidance mitigated the spread of COVID-19, but there was uneven application of this guidance initially | 5-6 |
|  | Variance in response and decision-making was a result of local-level commanders being given latitude to adjust response based on local conditions | 6 |
|  | Guidelines were focused on continental US installations, creating difficulties for outside of continental US installations | 6 |
|  | No single case tracking and reporting system existed across the DoD | 6 |
|  | Roles and responsibilities for biosurveillance were not clearly defined leading to suboptimal coordination | 6 |
|  | Key personnel did not consistently have defined roles and responsibilities, and execution of such authorities was sometimes precluded by local guidance | 6 |
|  | Air Force management structures were updated to create more consistent responsibilities in public health roles | 7 |
|  | A gap was identified for coordination of Public Health Emergency Management responses at the theater, command center, Combatant Command , and global levels | 7 |
|  | No clearly defined policy, plans, or training for contact tracing during a pandemic | 7 |
|  | At the start of the pandemic, research rapidly shifted to COVID-19 research, causing some non-COVID-19 research to expire or be lost | 7 |
|  | Legal and privacy issues created challenges surrounding vaccines | 8 |
|  | Physical safety measures were rapidly developed and deployed, helping to mitigate the spread of the virus in controlled military environments | 8 |
|  | Rapid deployment of forces across the Indo-Pacific Command did not have adequate pre-deployment protocols | 9 |
|  | Maintaining a Common Operating Procedure across multiple commands and agencies with conflicting perspectives was problematic | 9 |
|  | MHS rapidly shifted to telemedicine with increased need, but no unified strategy has been implemented | 9 |
|  | The overall MHS strategy for the use of virtual health solutions across the MHS has yet to be fully implemented | 9 |
|  | Six sets of Clinical Practice Guidelines were produced, but communication and implementation were not consistent across the MHS | 10 |
|  | DHA proactively developed health benefit policy, reimbursement, and claims processing requirements to ensure access to care | 14-15 |
|  | DHA promulgated rulemaking to authorize coverage of costs related to trials and investigational drugs related to COVID-19 | 15 |
|  | Managed care support contractors deferred or manually paid claims and implement initiatives to update pricing systems to ensure COVID claims were properly tracked | 15 |
|  | Some TRICARE providers were double paid for vaccination services, and some managed care support contractors applied cost shares for vaccination, despite waiving of cost share requirements | 15 |
|  | TRICARE managed care support contractors paid providers to administer vaccines in a manner that did not meet CDC requirements | 15 |
|  | DoD moved emergency funding early to commands for execution, but execution was too slow | 18 |
|  | Policy decisions that restricted access to MTFs drove beneficiaries to the Private Sector Care network and TRICARE in unexpected numbers | 18 |
|  | TRICARE was slow to approve certain telehealth and other treatment options and did not clearly communicate new service options and cost shares to beneficiaries | 18 |
|  | DHA created information toolkits to facilitate consistent and authoritative messaging to the private sector care network | 18 |
|  | DHA/TRICARE had issues with requests for exceptions to policy and clarifications’ being handled through unofficial channels | 18 |
|  | The limited period of availability for pandemic response funding adversely affected the efficient use of resources | 19 |
|  | The use of the provisions of the Economy Act, 31 U.S.C. § 1535, facilitated rapid exchange of funding, goods, and services between the NIH and elements of the MHS research enterprise through cooperative agreements | 19 |
